# Cinacalcet-induced hypocalcemia in a cohort of European haemodialysis patients: predictors, therapeutic approaches and outcomes

**DOI:** 10.1007/s40620-019-00686-z

**Published:** 2019-12-17

**Authors:** Karly S. Louie, Clement Erhard, David C. Wheeler, Peter Stenvinkel, Bruno Fouqueray, Jürgen Floege

**Affiliations:** 1 Division of Nephrology & Clinical Immunology, RWTH University of Aachen, Pauwelsstraẞe 30, 52057 Aachen, Germany; 2 Amgen Ltd., Uxbridge, UK; 3 Stanislas Limited, Uxbridge, UK; 4 Department of Nephrology, University College London, London UK and George Institute for Global Health, Sydney, Australia; 5 Division of Renal Medicine, Department of Clinical Science Technology and Intervention, Karolinska University Hospital, Karolinska Institute, Stockholm, Sweden; 6 Amgen GmbH, Rotkreuz, Switzerland

**Keywords:** Cinacalcet, Secondary hyperparathyroidism, Parathyroid hormone, Hypocalcemia, Hemodialysis, Calcimimetic

## Abstract

**Background:**

Calcimimetic treatment of secondary hyperparathyroidism in chronic dialysis patients is often followed by hypocalcemia.

**Methods:**

We investigated the frequency, predictors, consequences and therapeutic responses following cinacalcet-induced hypocalcemia in an incident European hemodialysis cohort of 1068 patients with a cinacalcet prescription.

**Results:**

Of 905 normocalcemic patients initiating cinacalcet, 67% developed hypocalcemia within 12 months: 68% mild, 23% moderate, 9% severe. Compared to persistently normocalcemic patients, those with severe hypocalcemia were more often diabetic, overweight, had cardiovascular disease, shorter dialysis vintage, used a catheter dialysis access, had fewer active vitamin-D sterols, and exhibited higher CRP and iPTH and lower calcium levels. Multivariate predictors of hypocalcemia included a catheter for vascular access, low albumin and high iPTH. Generally, no therapeutic intervention to prevent hypocalcemia was taken prior to cinacalcet initiation. After the hypocalcemic event, the most common clinical response was no change of the dialysis or medical regimen. Following the hypocalcemic event, iPTH remained low even in those with severe hypocalcemia. The number of deaths and cardiovascular events did not differ between patients with and without hypocalcemia within six months following cinacalcet initiation.

**Conclusion:**

Two-thirds of cinacalcet initiated patients experienced hypocalcaemia with 9% being severe. Hypocalcemia was mostly asymptomatic, transient (with and without targeted intervention to correct it) and not associated with an increase in cardiovascular events or deaths.

**Electronic supplementary material:**

The online version of this article (10.1007/s40620-019-00686-z) contains supplementary material, which is available to authorized users.

## Introduction

Secondary hyperparathyroidism (sHPT) is common in patients on chronic hemodialysis (HD). While some degree of parathyroid hormone (PTH) elevation is considered necessary to maintain normal bone turnover, about 10–20% of dialysis patients have excessively high PTH levels associated with high turnover bone disease, fractures, hypercalcemia episodes and increased mortality [1]. As recommended by the Kidney Disease: Improving Global Outcomes (KDIGO) chronic kidney disease-mineral bone disorder (CKD-MBD) Guideline, therapeutic approaches for dialysis patients with sHPT who have PTH levels above the target range include calcimimetics, calcitriol and active vitamin D sterols, or, in advanced cases, parathyroidectomy [2]. Elevated serum phosphate levels are often difficult to normalize despite dietary advice, prescription of phosphate binders and adjustment of dialysis regimens. Use of active vitamin D sterols for patients with advanced sHPT can lead to hypercalcemia and/or exacerbation of hyperphosphatemia. Thus, calcimimetics, such as cinacalcet, may be the treatment of choice since they do not increase serum calcium or phosphate in contrast to active vitamin D sterols. Moreover, calcimimetics markedly reduce FGF23, a phosphaturic hormone associated with cardiovascular mortality in dialysis patients [3].

In chronic HD patients with severe sHPT, the initiation of cinacalcet is frequently followed by reductions in serum calcium and phosphate that can last one year or longer [4–9]. This has been interpreted as a medically induced equivalent of the “hungry bone syndrome” [10] that is observed after parathyroidectomy for those with advanced sHPT. It is due to rapid and often pronounced incorporation of calcium and phosphate into a previously demineralized bone. Compared to the “hungry bone syndrome”, cinacalcet-induced hypocalcemia is usually milder, more prolonged and usually asymptomatic [4–8, 11]. The asymptomatic nature of cinacalcet induced hypocalcemia was a central reason for the 2017 revision of the KDIGO CKD-MBD guideline that recommends avoidance of hypercalcemia, whereas mild hypocalcemia was considered acceptable, particularly if it is associated with calcimimetic use [2]. Presently it is uncertain whether calcimimetic induced hypocalcemia requires clinical interventions to increase calcium levels in chronic HD patients [12].

To complement findings from the large randomized controlled Evaluation Of Cinacalcet Hydrochloride Therapy to Lower CardioVascular Events (EVOLVE) trial where the majority of patients with hypocalcemia resolved spontaneously within 14 days without any therapeutic intervention [11], we assessed cinacalcet-induced hypocalcemia in a large incident European HD cohort in a “real-world” setting. The study objectives were (a) to describe the incidence of hypocalcemia in the 12 months following cinacalcet initiation, (b) to describe discontinuation and re-initiation of cinacalcet following first hypocalcemia episode (Ca < 2.1 mmol/L), (c) to describe factors associated with hypocalcemia, (d) to describe treatment patterns prior to cinacalcet initiation and after hypocalcemia episode; and (e) to describe the number of cardiovascular events and deaths among those who developed hypocalcemia and no hypocalcemia.

## Methods

### Study design

The Analysing Data, Recognising Excellence and Optimising Outcomes research initiative phase 2 (AROii) is a retrospective longitudinal cohort of incident HD patients (< 6 months on dialysis), who have not undergone prior kidney transplantation, who attended one of > 300 Fresenius Medical Care (FMC) facilities in 14 European countries (Czech Republic, France, Hungary, Ireland, Italy, Poland, Portugal, Romania, Russia, Serbia, Slovak Republic, Slovenia, Spain, Turkey and the United Kingdom). Methods used in analysis of the AROii cohort have been described previously [13, 14]. Longitudinal anonymised individual-level data on medical history (hospitalisation, diabetes, cancer, cardiovascular disease and facture), laboratory (albumin, calcium, c-reactive protein (CRP), ferritin, hemoglobin, phosphate, and PTH), dialysis (frequency, duration per week, dialysis adequacy, dialysis vintage, dialysis calcium, catheter vascular access, and net ultrafiltration), and medication data (calcitriol/alfacalcidol, paricalcitol, phosphate binders), plus ICD-10 coded hospitalisation and death data are available for patients who enrolled in AROii between 1 January 2007 and 31 December 2009 and were followed-up to end of 2014. Data were collected and processed in accordance with local ethical and regulatory requirements for each participating FMC facility. Written informed consent was obtained from all patients, where required.

### Study population

Chronic HD patients (> 10 contiguous dialysis sessions) aged ≥ 18 who received a cinacalcet prescription after 1 January 2007, were enrolled in AROii for at least 90 days prior to cinacalcet initiation and had at least 90 days of follow-up after initiation were eligible for study inclusion. Patients who had undergone parathyroidectomy up to and including the first 90 days of follow-up or who had no further cinacalcet prescriptions or who had further prescriptions of less than 15 days were excluded.

The index date was date of cinacalcet initiation. The baseline period was defined as the 90 days prior to cinacalcet initiation. Patients were followed up for 12 months following cinacalcet initiation to determine the incidence of hypocalcemia. Then patients with hypocalcemia were followed-up for an additional 4-months to describe cinacalcet continuation and to describe treatment and management of patients following the episode. Among those who discontinued cinacalcet within the 4-month period, the probability of re-initiation was described in the 12-months following discontinuation. Patients were censored if they died, underwent parathyroidectomy, had a renal transplant, or were lost to follow-up. Patients were considered lost to follow-up if they left a dialysis facility for any reason and did not return to an FMC facility within 45 days.

The main cohort for analysis was restricted to patients with normal uncorrected total serum calcium levels ≥ 2.1 mmol/L at time of cinacalcet initiation. In clinical practice, physicians use uncorrected or albumin-corrected calcium to evaluate hypocalcemia although data suggest that uncorrected is not inferior to corrected calcium [15, 16]; therefore, sensitivity analyses were also carried out based with albumin-corrected calcium. Similar to previous studies, hypocalcemia was defined as mild (calcium 2.0− < 2.1 mmol/L), moderate (calcium 1.87− < 2.0 mmol/L), and severe (calcium < 1.87 mmol/L).

Treatment characteristics (cinacalcet dose, calcitriol/alfacalcidol, paricalcitol, calcium-based phosphate binder, dialysate Ca) were summarized before cinacalcet initiation and after the hypocalcemia episode. Patients were classified as new users or continuing users and their dose change (up-titration, down-titration). For the period before cinacalcet initiation, patients were classified as continuing users if they had a continuous prescription − 60 days up to index date. Dose change was classified as up-titrated if the average daily dose between − 30 days and index date was greater than the average daily dose between − 60 and − 31 days before index date; and down-titrated if the average daily dose between − 30 and index date was less than the average daily dose between − 60 and − 31 days before index date; and stable dose if the average daily dose between − 30 days and index date equaled the average daily dose between − 60 and − 31 days before the index date. Patients were classified as new users if they had a prescription between − 30 days and the index date but there was no prescription between − 60 and − 31 days before index date. For the period after the hypocalcemia episode, treatment characteristics for the 30-days period after the hypocalcemia event (i.e., defined as the intervention period) was compared to the − 30 days period before hypocalcemia. The proportion of patients who achieved normal Ca levels following this treatment intervention period was described at 30, 60 and 90 days afterwards. Sensitivity analyses were also carried out for a subset of subjects with albumin-corrected calcium.

### Statistical analysis

Statistical analyses were conducted in SAS (version 9.4; SAS, Cary, NC, USA) and reproduced independently. Baseline characteristics of patients who developed hypocalcemia vs. no hypocalcemia were described using median and interquartile range for continuous variables and counts and proportions for categorical variable parameters. The most recent values available in the 90 days prior to the date of cinacalcet initiation (baseline period) for dialysis, laboratory and medication variables were summarised. To describe factors associated with first hypocalcemia episode in the first 12 months after cinacalcet initiation, Cox regression models were constructed. Variables significant at p < 0.25 level in univariate analysis were entered into a multivariate model using stepwise regression (significance level of p < 0.10 for inclusion/exclusion); and hazard ratios (HR) and 95% confidence intervals (CI) were calculated. To describe time-to-event (first hypocalcemia episode, cinacalcet discontinuation, and cinacalcet re-initiation), Kaplan–Meier curves were plotted, and cumulative monthly probabilities were estimated.

## Results

### Occurrence of hypocalcemia

**Fig. 1:**
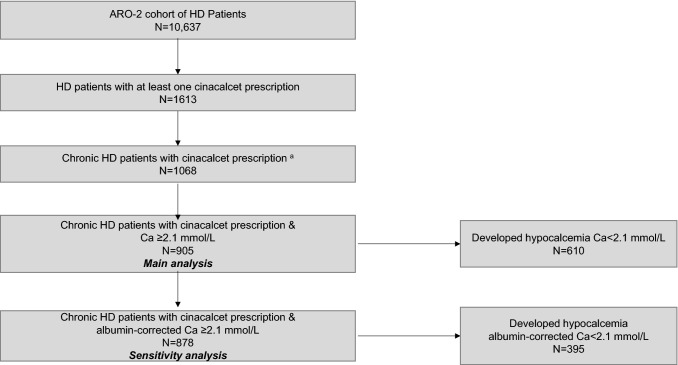
Study flow chart. ^a^Chronic HD patients in ARO-2 cohort aged ≥18, filled a cinacalcet prescription after 1 January 2007, enrolled in the cohort for at least 90 days prior to cinacalcet initiation and have at least 90 days follow-up following cinacalcet initiation. Twenty six patients did not meet inclusion criteria (13 patients had a parathyroidectomy and 33 patients only one prescription or no further prescriptions or had further prescriptions of less than 15 days) and were excluded.

Country-specific cinacalcet prescriptions varied widely ranging from 2% in Turkey and Romania to 35-39% of the HD patients in Spain and Ireland (Table 1). In the AROii cohort we identified 1068 HD patients with a cinacalcet prescription and 905 (84.7%) patients had normal (Ca ≥ 2.1 mmol/L) and 163 patients (15.3%) had hypocalcaemia (Ca < 2.1 mmol/L) at time of first cinacalcet prescription (Fig. 1). Of these 905 patients who had a normal total serum calcium ≥ 2.1 mmol/L at time of cinacalcet initiation, 610 (67%) subsequently developed hypocalcemia (i.e. serum calcium < 2.1 mmol/L) within 12 months of the initiation of the cinacalcet prescription. For the sensitivity analysis, of 878 patients (97%) had an albumin-corrected Ca ≥ 2.1 mmol/L value available at cinacalcet initiation, 395 (45%) subsequently developed hypocalcemia (Online Resource 1).

**Table 1:** Number of subjects with cinacalcet prescriptions in the AROii cohort

Country	Total no. of subjects in AROii cohort	Subjects with at least one prescription, n (%)
Czech Republic	688	66 (10)
France	92	17 (18)
Hungary	654	21 (3)
Ireland	23	9 (39)
Italy	561	62 (11)
Poland	97	7 (7)
Portugal	2319	443 (19)
Romania	514	10 (2)
Russia	102	10 (10)
Serbia	88	2 (2)
Slovak Republic	9	1 (11)
Slovenia	86	10 (12)
Spain	2429	852 (35)
Turkey	1760	27 (2)
United Kingdom	1215	76 (6)
Total	10,637	1613 (15)

**Fig. 2:**
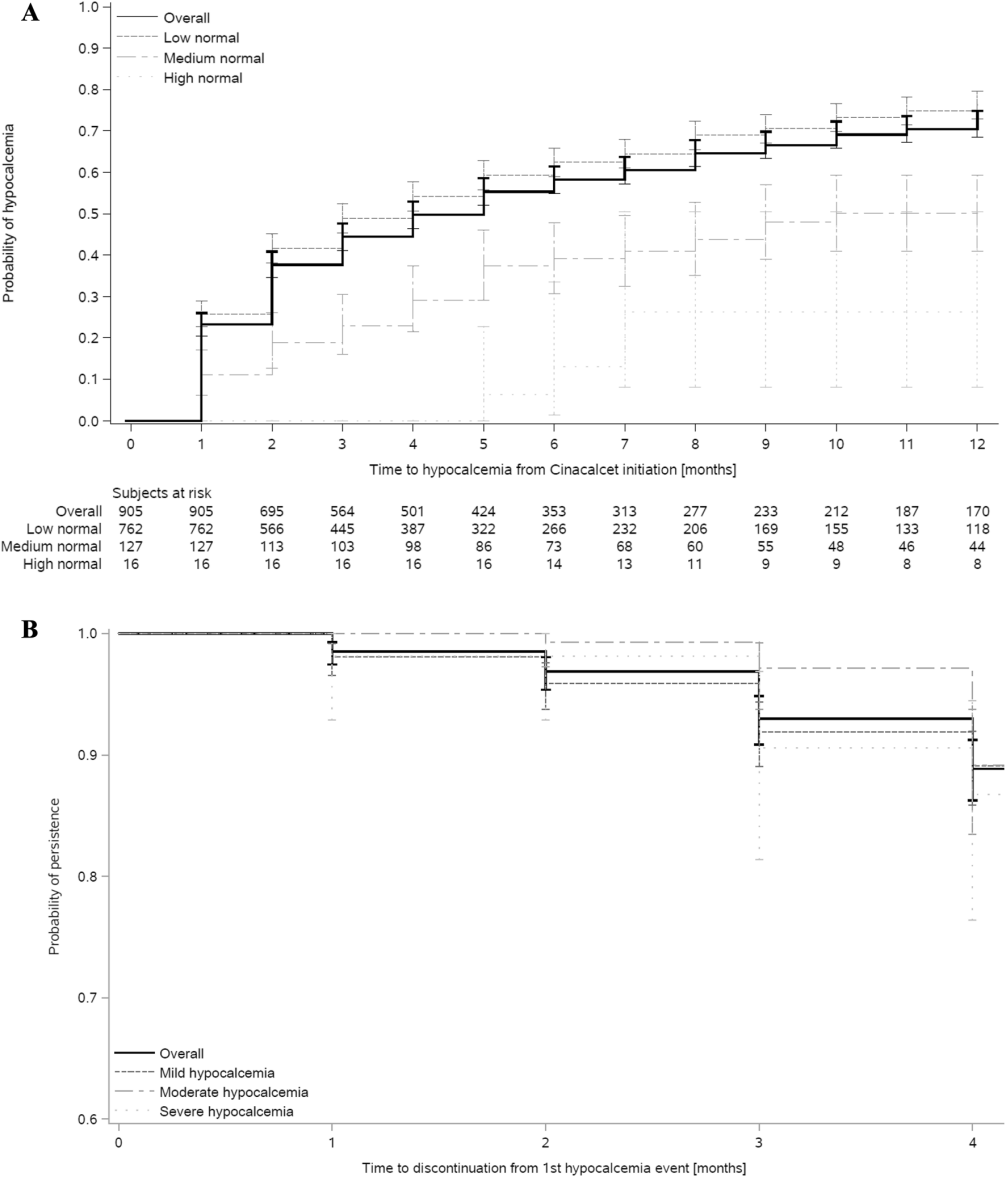
**a** Kaplan–Meier curve of time to hypocalcemia episode, overall and by total serum Ca at time of cinacalcet initiation. **b** Kaplan–Meier curve of time to cinacalcet discontinuation in the 4 months following hypocalcemia episode, overall and by total serum Ca at time of hypocalcemia episode

Figure 2a shows the cumulative probability of experiencing a hypocalcemic event (based on uncorrected calcium) at 6 and 12 months as 58% and 72%, respectively, after cinacalcet initiation. Manifestation of hypocalcemia was a function of the baseline calcium and less likely in the few patients with overt hypercalcemia. The 12-month cumulative probability for those who had low-levels of normal calcium (2.1– ≤ 2.5 mmol/L) compared to those who had hypercalcemia (calcium ≥ 2.75 mmol/L) was 76% and 26%, respectively. The median time to the first hypocalcemia episode was 4 months. However, according to corrected calcium, the cumulative probability of experiencing a hypocalcemia episode at 6 and 12 months was lower, 37% and 49%, respectively (Online Resource 2).

Of the 610 who developed hypocalcemia after cinacalcet initiation, 68% (n = 415) had mild, 23% (n = 141) had moderate, and 9% (n = 54) had severe hypocalcemia. Following the first hypocalcemic event, 89% of the patients were persistent with their cinacalcet prescription at four months (Fig. 2b). Continuation was similar in those who had mild and severe hypocalcemia, 89% vs. 87%, respectively. Similar persistence was also observed for those in the sensitivity analysis with hypocalcemia (corrected calcium) at four months: 88% overall, 89% for mild and 85% for severe hypocalcemia (Online Resource 3).

Among the 66 patients who discontinued cinacalcet within four months after the hypocalcemia episode, the cumulative probability of re-initiation at 12 months was 40% and 50% in the mild and moderate hypocalcemia groups, respectively (data not shown). This cumulative probability dropped to 20% in the seven patients with severe hypocalcemia. For the sensitivity analysis, the cumulative probability of re-initiation at 12 months was 38% and 56% for the mild and moderate groups and this decreased to 30% in the eight patients with severe hypocalcemia (data not shown).

### Characteristics of patients with and without hypocalcemia

Table 2 describes the characteristics of patients who developed hypocalcemia or no hypocalcemia within 12 months of cinacalcet initiation. Generally, characteristics were similar among the two groups. However, in assessing baseline demographics, patients with severe hypocalcemia as compared to those with no hypocalcemia were more likely to be diabetic (37% vs. 30%), overweight (body mass index ≥ 25 kg/m^2^, 64% vs. 59%), have prior cardiovascular disease (52% vs. 44%), have shorter dialysis vintage (1.7 vs. 2.0 years), more likely to use a dialysis catheter as access (46% vs. 13%), use fewer active vitamin-D sterols (54% vs. 68%), and exhibit higher median CRP (7.4 vs. 4.1 mg/L) and iPTH (677 vs. 545 ng/L) and lower median uncorrected calcium levels (2.3 vs. 2.4 mmol/L). In contrast, when compared to patients with no hypocalcemia according to corrected calcium, (Online Resource 1), severely hypocalcemic patients were younger, more likely to be diabetic, obese, have less cardiovascular disease, similar dialysis vintage, more likely to have a dialysis catheter access, had higher phosphate levels, were more likely to be using active vitamin-D sterols and less likely to be using calcium-based phosphate binders.

**Table 2:** Baseline characteristics of patients who developed and did no develop hypocalcemia within 12 months after cinacalcet initiation

Characteristic	No hypocalcemia N = 295	Hypocalcemia N = 610	Patients who develop hypocalcemia within 12 months (N = 610)		
			Mild Ca 2.0– < 2.1 mmol/L N = 415	Moderate Ca 1.87– < 2.0 mmol/L N = 141	Severe Ca < 1.87 mmol/L N = 54
Patient age at index date [years]^a^	66 (54, 75)	67 (54, 76)	67 (54, 77)	66 (55, 75)	69 (60, 77)
Male, no. (%)	173 (59)	352 (58)	240 (58)	82 (58)	30 (56)
Geographical area, no. (%)					
Eastern Europe^b^	25 (9)	56 (9)	38 (9)	15 (11)	3 (6)
Western Europe^c^	33 (11)	41 (7)	28 (7)	8 (6)	5 (9)
Iberian Peninsula^d^	237 (80)	513 (84)	349 (84)	118 (84)	46 (85)
BMI [kg/m^2^]^a^	26.9 (24.0, 31.0)	27.0 (24.3, 30.2)	26.9 (24.2, 30.2)	27.2 (24.3, 30.3)	27.5 (25.5, 30.0)
BMI [kg/m^2^]—category, No. (%)					
< 18.5	4 (1)	7 (1)	6 (1)	1 (1)	0 (0)
≥ 18.5–< 25	79 (27)	162 (27)	115 (28)	37 (26)	10 (19)
≥ 25–< 30	101 (34)	232 (38)	154 (37)	54 (38)	24 (44)
≥ 30	75 (25)	148 (24)	102 (25)	35 (25)	11 (20)
Missing	36 (12)	61 (10)	38 (9)	14 (10)	9 (17)
Clinical history, no. (%)					
Hospitalisation	137 (46)	310 (51)	207 (50)	71 (50)	32 (59)
Diabetes	89 (30)	194 (32)	128 (31)	46 (33)	20 (37)
Cancer	26 (9)	59 (10)	42 (10)	11 (8)	6 (11)
Cardiovascular disease	131 (44)	304 (50)	199 (48)	77 (55)	28 (52)
Fracture	14 (5)	29 (5)	21 (5)	6 (4)	2 (4)
Dialysis duration per week [hours]^a^	12.0 (12.0, 12.4)	12.0 (12.0, 12.3)	12.0 (12.0, 12.3)	12.0 (12.0, 12.4)	12.0 (12.0, 12.0)
Dialysis adequacy [Kt/V]–category, no. (%)	39 (13)	88 (14)	53 (13)	23 (16)	12 (22)
< 1.2	229 (78)	474 (78)	320 (77)	112 (79)	42 (78)
≥ 1.2	27 (9)	48 (8)	42 (10)	6 (4)	0 (0)
Dialysis vintage [years]^a^	2.0 (1.0, 3.5)	1.8 (1.0, 3.0)	1.8 (1.0, 3.1)	1.8 (0.9, 3.0)	1.7 (0.9, 2.3)
Dialysate calcium [mmol/L]^a^	1.3 (1.3, 1.5)	1.3 (1.3, 1.5)	1.3 (1.3, 1.5)	1.3 (1.3, 1.5)	1.5 (1.3, 1.5)
Catheter vascular access, no. (%)					
Yes	37 (13)	158 (26)	99 (24)	34 (24)	25 (46)
No	246 (83)	434 (71)	306 (74)	101 (72)	27 (50)
Missing	12 (4)	18 (3)	10 (2)	6 (4)	2 (4)
Net ultrafiltration [L]^a^	2.0 (1.4, 2.7)	2.1 (1.4, 2.7)	2.0 (1.4, 2.7)	2.1 (1.5, 2.6)	2.3 (1.7, 3.0)
Blood haemoglobin [g/L]^a^	118 (110, 126)	120 (112, 127)	119 (111, 127)	121 (112, 127)	119 (113, 127)
Serum albumin [g/L]^a^	41.0 (38.4, 43.0)	40.0 (37.0, 42.2)	40.0 (37.9, 42.1)	39.2 (37.0, 43.0)	38.4 (36.0, 42.0)
Serum ferritin [µg/L]^a^	430 (244, 639)	398 (260, 599)	410 (267, 620)	382 (242, 544)	376 (236, 572)
Serum CRP [mg/L]^a^	4.1 (1.7, 9.2)	4.9 (2.0, 11.0)	4.7 (1.8, 10.0)	5.0 (2.0, 12.3)	7.4 (2.8, 17.7)
Serum calcium [mmol/L]^a^	2.4 (2.3, 2.5)	2.3 (2.2, 2.4)	2.3 (2.2, 2.4)	2.3 (2.2, 2.4)	2.3 (2.2, 2.3)
Serum calcium [mmol/L]—category, no. (%)					
≥ 2.10–< 2.50	216 (73)	546 (90)	367 (88)	131 (93)	48 (89)
≥ 2.50–< 2.75	67 (23)	60 (10)	44 (11)	10 (7)	6 (11)
≥ 2.75	12 (4)	4 (1)	4 (1)	0 (0)	0 (0)
Serum phosphate [mmol/L]^a^	1.7 (1.4, 2.0)	1.7 (1.4, 2.0)	1.7 (1.4, 2.0)	1.7 (1.5, 2.0)	1.7 (1.3, 1.9)
Serum PTH [ng/L]^a^	545 (415, 761)	606 (442, 837)	580 (434, 833)	624 (446, 864)	677 (463, 824)
Corrected Ca [mmol/L]^a^	2.4 (2.3, 2.5)	2.3 (2.2, 2.4)	2.3 (2.3, 2.4)	2.3 (2.2, 2.4)	2.3 (2.2, 2.4)
Corrected Ca [mmol/L], no. (%)					
≥ 2.10–< 2.50	190 (64)	504 (83)	342 (82)	119 (84)	43 (80)
≥ 2.50–< 2.75	73 (25)	68 (11)	47 (11)	14 (10)	7 (13)
≥ 2.75	14 (5)	4 (1)	4 (1)	0 (0)	0 (0)
Missing	18 (6)	34 (6)	22 (5)	8 (6)	4 (7)
Drug use, no. (%)					
Calcitriol/alfacalcidol	75 (25)	149 (24)	98 (24)	41 (29)	10 (19)
Paricalcitol	127 (43)	265 (43)	186 (45)	60 (43)	19 (35)
Phosphate binder use, no. (%)					
Non-calcium based only	136 (46)	257 (42)	187 (45)	50 (36)	20 (37)
Calcium based only	26 (9)	66 (11)	41 (10)	19 (14)	6 (11)
Both calcium based and non-calcium based	71 (24)	137 (23)	96 (23)	31 (22)	10 (19)
None	62 (21)	150 (25)	91 (22)	41 (29)	18 (33)

^a^Median (Q1, Q3)

^b^Eastern Europe: Czech Republic, Hungary, Poland, Romania, Russia, Serbia, Slovak Republic, Slovenia and Turkey

^c^Western Europe: France, Ireland, Italy, and the United Kingdom

^d^Iberian Peninsula: Portugal and Spain

### Baseline correlates of hypocalcemia

Table 3 shows that in multivariate analysis, baseline predictors for first hypocalcemia episode within 12 months after starting cinacalcet were significant for geographical origin of the patients, having a catheter vascular access, low albumin and high iPTH. Low haemoglobin was associated with a lower risk of hypocalcemia. Table 4 shows significant predictors of severe hypocalcemia which were having a catheter access, high net ultrafiltration, high CRP, and no paricalcitol use. Online Resource 4 and Online Resource 5 describe predictors according to hypocalcemia as defined by corrected calcium. Predictors were generally similar for hypocalcemia vs. no hypocalcemia; whereas having a catheter access was a strong predictor for severe hypocalcemia.

**Table 3:** Baseline predictors of time to first hypocalcemia episode in the first 12 months after cinacalcet initiation

Characteristics	Any hypocalcemia vs no hypocalcemia		
	No. of hypocalcemia events/total no. of subjects (%)	Hazard ratio (95% CI)	p value
Geographical area			0.01
Iberian Peninsula^a^	513/750 (68.4)	References	
Eastern Europe^b^	56/81 (69.1)	1.22 (0.87, 1.70)	
Western Europe^c^	41/74 (55.4)	0.65 (0.46, 0.90)	
Catheter vascular access			<0.001
No	434/680 (63.8)	References	
Yes	158/195 (81.0)	1.59 (1.32, 1.92)	
Missing information	18/30 (60.0)	0.91 (0.52, 1.57)	
Haemoglobin [g/dL]			0.03
≥ 12	306/436 (70.2)	References	
10–< 12	276/423 (65.2)	0.87 (0.74, 1.03)	
< 10	28/46 (60.9)	0.61 (0.41, 0.91)	
Serum albumin [g/L]			0.003
Q4	98/160 (61.3)	References	
Q3	155/258 (60.1)	1.07 (0.83, 1.37)	
Q2	167/222 (75.2)	1.46 (1.13, 1.87)	
Q1	156/213 (73.2)	1.47 (1.14, 1.91)	
Missing data	34/52 (65.4)	1.25 (0.84, 1.85)	
PTH [pg/mL]			0.005
< 600	303/476 (63.7)	References	
600–< 1000	206/283 (72.8)	1.32 (1.11, 1.58)	
≥ 1000	101/146 (69.2)	1.26 (0.99, 1.60)	

*Q* quartile

^a^Eastern Europe: Czech Republic, Hungary, Poland, Romania, Russia, Serbia, Slovak Republic, Slovenia and Turkey

^b^Western Europe: France, Ireland, Italy, and the United Kingdom

^c^Iberian Peninsula: Portugal and Spain

**Table 4:** Baseline predictors of time to first severe hypocalcemia episode (Ca < 1.87 mmol/L)

	Severe hypocalcemia vs. No hypocalcemia		
	No. of patients with severe hypocalcemia/ Total no. of subjects	Hazard ratio (95% CI)	p-value
Catheter vascular access			<0.001
No	27/273 (9.9)	References	
Yes	25/62 (40.3)	5.72 (3.23, 10.13)	
Missing	2/14 (14.3)	1.07 (0.24, 4.79)	
Net Ultrafiltration [L]			0.004
Q1	7/89 (7.9)	References	
Q2	17/85 (20.0)	4.73 (1.88, 11.93)	
Q3	12/87 (13.8)	2.29 (0.88, 5.93)	
Q4	18/88 (20.5)	4.16 (1.68, 10.28)	
CRP [mg/L]			0.04
Q1	3/60 (5.0)	References	
Q2	12/76 (15.8)	5.18 (1.42, 18.82)	
Q3	7/63 (11.1)	2.87 (0.73, 11.31)	
Q4	17/63 (27.0)	5.76 (1.68, 19.75)	
Missing	15/87 (17.2)	3.32 (0.93, 11.84)	
Paricalcitol use			0.04
Yes	19/146 (13.0)	References	
No	35/203 (17.2)	1.81 (1.02, 3.24)	

*CRP* creatinine reactive protein, *Q* quartile

### Medical therapy immediately prior to the first hypocalcemia episode

To evaluate whether there was any prior intervention to prevent hypocalcemia before initiating cinacalcet, we compared the medical regimen at the time of first hypocalcemia. Generally, no intervention was taken prior to start of cinacalcet prescription. As shown in Table 5, patients developing hypocalcemia vs. no hypocalcemia received a similar dosage of cinacalcet at initiation (median average daily dose 30 mg), similar and stable usage of active vitamin D sterols, and had a similar and stable dialysate calcium concentration. Calcium-containing phosphate binders were used less frequently in patients with high baseline serum calcium, who subsequently developed hypocalcemia. Findings were relatively similar in the sensitivity analysis (Online Resource 6).

**Table 5:** Treatment characteristics of chronic haemodialysis patients at time of cinacalcet initiation

	No hypocalcemia within 12 months after cinacalcet initiation N = 295	Hypocalcemia within 12 months after cinacalcet initiation		
		Ca at time of cinacalcet initiation		
		Overall Ca ≥ 2.1 mmol/L N = 610	Low normal Ca 2.10– < 2.50 mmol/L N = 546	Moderate to high normal Ca ≥ 2.5 mmol/L N = 64
Cinacalcet				
Average daily dose [mg/day], median (Q1, Q3)	30.0 (12.9, 30.0)	30.0 (17.1, 30.0)	30.0 (17.1, 30.0)	30.0 (30.0, 30.0)
Calcitriol/alfacalcidol				
Use, n (%)	60 (20)	130 (21)	116 (21)	14 (22)
Daily dose [µg/day], median (Q1, Q3)	0.4 (0.2, 0.5)	0.3 (0.2, 0.4)	0.3 (0.2, 0.4)	0.3 (0.2, 0.6)
New users, n (%)	2 (3)	4 (3)	4 (3)	0 (0)
Continuing users, n (%)	58 (97)	126 (97)	112 (97)	14 (100)
Stable dose, n (%)	47 (81)	106 (84)	95 (85)	11 (79)
Up-titration, n (%)	7 (12)	9 (7)	9 (8)	0 (0)
Down-titration, n (%)	4 (7)	11 (9)	8 (7)	3 (21)
Paricalcitol				
Use, n (%)	113 (38)	234 (38)	207 (38)	27 (42)
Daily dose [µg/day], median (Q1, Q3)	0.9 (0.6, 1.4)	0.9 (0.6, 1.2)	0.9 (0.6, 1.1)	0.9 (0.7, 1.9)
New users, n (%)	2 (2)	17 (7)	17 (8)	0 (0)
Continuing users, n (%)	111 (98)	217 (93)	190 (92)	27 (100)
Stable dose, n (%)	78 (70)	164 (76)	146 (77)	18 (67)
Up-titration, n (%)	23 (21)	26 (12)	21 (11)	5 (19)
Down-titration, n (%)	10 (9)	27 (12)	23 (12)	4 (15)
Calcium-based phosphate binder				
Use, n (%)	64 (22)	140 (23)	132 (24)	8 (13)
Daily dose [mg/day], median (Q1, Q3)	1943 (715, 2820)	1980 (1143, 3150)	1980 (1143, 3000)	2133 (1240, 4600)
New users, n (%)	1 (2)	6 (4)	6 (5)	0 (0)
Continuing users, n (%)	63 (98)	134 (96)	126 (95)	8 (100)
Stable dose, n (%)	56 (89)	120 (90)	113 (90)	7 (88)
Up-titration, n (%)	4 (6)	8 (6)	8 (6)	0 (0)
Down-titration, n (%)	3 (5)	6 (4)	5 (4)	1 (13)
Dialysate calcium [mmol/L]				
Missing	11 (4)	18 (3)	14 (3)	4 (6)
≤ 1.00	1 (0)	2 (0)	2 (0)	0 (0)
> 1.00– < 1.25	0 (0)	0 (0)	0 (0)	0 (0)
≥ 1.25– < 1.50	146 (49)	306 (50)	271 (50)	35 (55)
≥ 1.50	137 (46)	284 (47)	259 (47)	25 (39)
Stable concentration, n (%)	264 (93)	552 (94)	497 (94)	55 (92)
Up-titration, n (%)	8 (3)	13 (2)	13 (2)	0 (0)
Down-titration, n (%)	12 (4)	24 (4)	19 (4)	5 (8)

### Medical therapy, PTH and cardiovascular episodes after the first hypocalcemia episode

Table 6 shows that the most common response to first hypocalcemia within 90 days following detection was no change to the therapeutic regimen with respect to dialysate calcium concentration, or dose of cinacalcet, active vitamin D and calcium containing phosphate binder. Similar observations were made when we assessed responses at 30 and 60 days (data not shown). Findings were similar in the sensitivity analysis (Online Resource 7).

**Table 6:** Management of hypocalcemia within 90 days following first hypocalcemia episode

Management of hypocalcemia following first hypocalcemic event	Overall			First hypocalcemia								
				Mild Ca 2.0– < 2.1 mmol/L N = 415			Moderate Ca 1.87– < 2.0 mmol/L N = 141			Severe Ca < 1.87 mmol/L N = 54		
	No. at risk	n	%	No. at risk	n	%	No. at risk	n	%	No. at risk	n	%
Dialysate calcium concentration												
Increased	610	102	17	415	60	15	141	30	21	54	12	22
Stable	610	472	77	415	329	79	141	103	73	54	40	74
Decreased	610	15	3	415	13	3	141	2	1	54	0	0
Cinacalcet												
Increased	610	91	15	415	67	16	141	21	15	54	3	6
Stable	610	374	61	415	255	61	141	86	61	54	33	61
Discontinued	610	42	7	415	33	8	141	4	3	54	5	9
Reduced	610	102	17	415	59	14	141	30	21	54	13	24
Calcitriol/alfacalcidol												
Initiated	486	41	8	326	20	6	115	13	11	45	8	18
Up-titrated	124	30	24	89	23	26	26	5	19	9	2	22
Initiated or up-titrated	610	71	12	415	43	10	141	18	13	54	10	19
Stable	124	73	59	89	53	60	26	14	54	9	6	67
Down-titrated	124	17	14	89	11	12	26	5	19	9	1	11
Discontinued	124	4	3	89	2	2	26	2	8	9	0	0
Paricalcitol												
Initiated	394	65	17	258	39	15	96	18	19	40	8	20
Up-titrated	216	54	25	157	40	26	45	11	24	14	3	21
Initiated or up-titrated	610	119	20	415	79	19	141	29	21	54	11	20
Stable	216	115	53	157	84	54	45	22	49	14	9	64
Down-titrated	216	34	16	157	24	15	45	8	18	14	2	14
Discontinued	216	13	6	157	9	6	45	4	9	14	0	0
Calcium-based phosphate binder												
Initiated	459	51	11	311	30	10	107	17	16	41	4	10
Up-titrated	151	25	17	104	14	14	34	9	27	13	2	15
Initiated or up-titrated	610	76	13	415	44	11	141	26	18	54	6	11
Stable	151	106	70	104	78	75	34	18	53	13	10	77
Down-titrated	151	14	9	104	8	8	34	5	15	13	1	8
Discontinued	151	6	4	104	4	4	34	2	6	13	0	0
Any expected responses^1^												
No treatment intervention	610	255	42	415	188	45	141	51	36	54	16	30
Any treatment intervention	610	355	58	415	227	55	141	90	64	54	38	70
Any responses^2^												
No treatment intervention	610	175	29	415	130	31	141	33	23	54	12	22
Any treatment intervention	610	435	71	415	285	69	141	108	77	54	42	78

^1^Any expected treatment response = for Cinacalcet, discontinued or reduced; for Dialysate Calcium: increased calcium; for other drugs: initiated or up-titrated

^2^Any treatment = any of the changes listed in table

About one-third (38%) of patients with a hypocalcemia episode had a therapeutic intervention (i.e. discontinued or reduced cinacalcet, increased dialysate calcium or increased vitamin D or calcium-based phosphate binders) within the first 30 days after the hypocalcemia event. This increased to 44% for those who had an episode of severe hypocalcemia (data not shown). Both groups of patients who had a treatment intervention or no intervention were able to achieve a normal calcium at days 30 (after intervention: 43% vs. none: 38%), 60 (64% vs. 60%) and 90 (73% vs. 75%) after the therapeutic intervention period. Findings were similar in the sensitivity analysis (data not shown). When examining the baseline characteristics of hypocalcemia patients who discontinued and did not discontinue within four months following hypocalcemia events, characteristics were relatively similar except baseline PTH at time of cinacalcet initiation was lower among those who discontinued. This suggests that discontinuation was largely dependent on PTH rather than hypocalcemia (Online Resource 8).

**Fig. 3:**
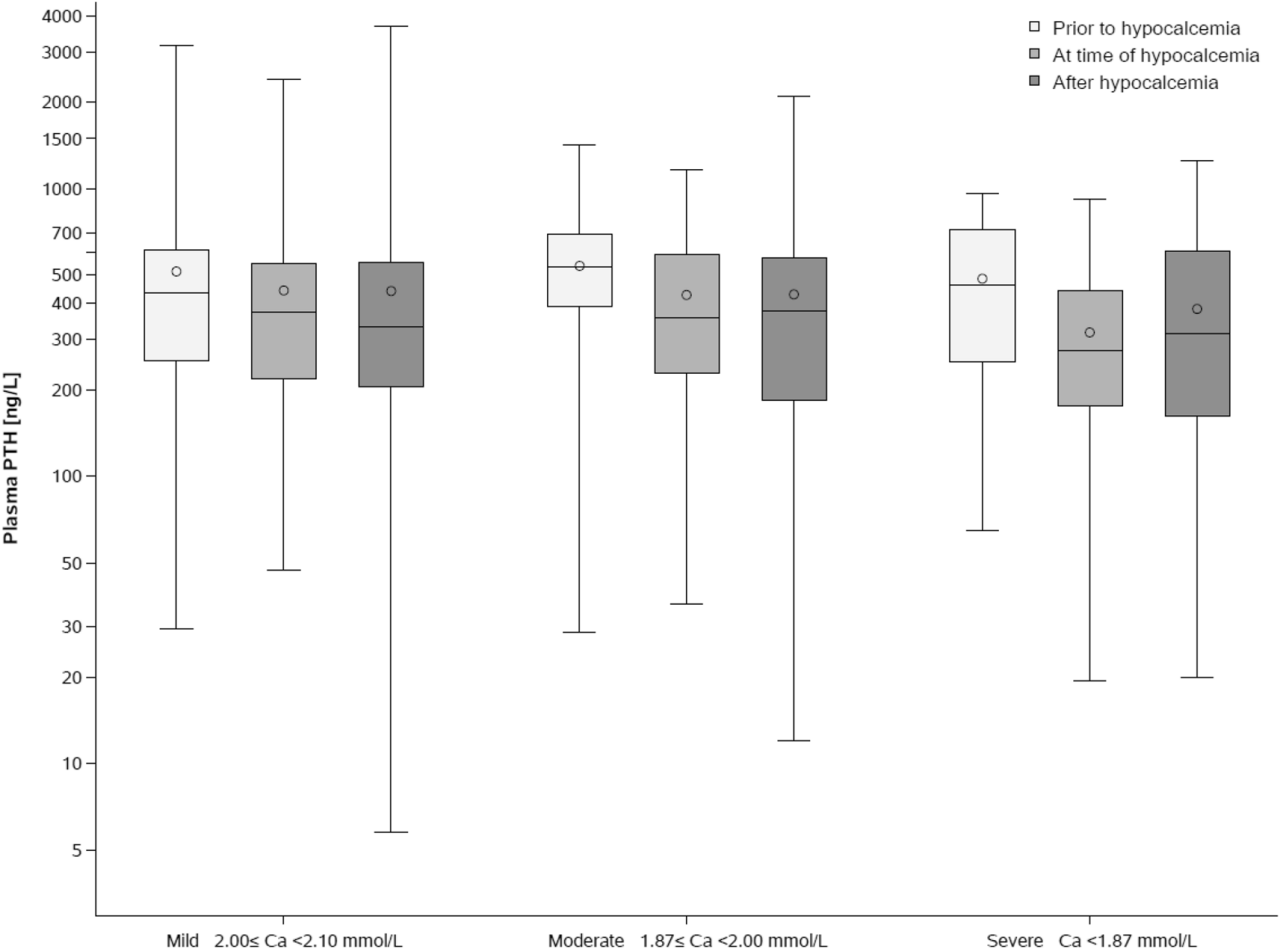
Box plot of PTH levels before, at and after first hypocalcemia episode by severity

Figure 3 shows closest available iPTH values before, at and after a hypocalcemia episode. No notable changes were observed. iPTH values at time of a hypocalcemia episode were lower than values prior to the episode. Following the hypocalcemia episode, iPTH stayed low even in those in the severe hypocalcemia group. Table 7 shows that the number of deaths and cardiovascular events did not differ in patients who did and did not develop hypocalcemia within 12 months following cinacalcet initiation.

**Table 7:** Number of patients with at least one cardiovascular event or death within 6 months after cinacalcet initiation

Definition of cardiovascular events and death	Hypocalcemia within 6 months after cinacalcet initiation N = 610		No hypocalcemia within 6 months after cinacalcet initiation N = 295	
	No. of events within 6 months following cinacalcet initiation	%	No. of events within 6 months following cinacalcet initiation	%
Any cardiovascular event^1^	34	5.6	16	5.4
Coronary artery events^1,2^	12	2.0	5	1.7
Cerebrovascular events^1,3^	4	0.7	4	1.4
Peripheral artery events^1,4^	10	1.6	4	1.4
Congestive heart failure^1,5^	9	1.5	1	0.3
Sudden cardiac death^1,6^	1	0.2	3	1.0
Deaths	8	1.3	9	3.1

^1^Cardiovascular events as defined in [27]

^2^Coronary artery events: ICD-10 code I20–I25

^3^Cerebrovascular events: ICD-10 codes F01, G45, G46, I60, I61, I62, I63, I64, I65, I66, I67, I68, I69

^4^Peripheral arterial events: ICD-10 codes H34, I51.3, I70, I71, I73.1, I73.9, I74, K55.0, K55.1, N28.0, R02

^5^Congestive heart failure events: ICD-10 codes I11.0, I13.0, I13.2, I42, I43, I50, I51.5, I51.7

^6^Sudden cardiac death: ICD-10 codes I46, I49.0

## Discussion

In this “real-world” analysis of a large European cohort of chronic HD patients we found that 67% of those initiating cinacalcet subsequently developed hypocalcemia. This frequency is similar to our recent post hoc analysis of the EVOLVE randomized controlled trial, in which cinacalcet-induced hypocalcemia was detected in 58% of patients within a median of 56 days after cinacalcet initiation [11]. This study found that 9% of the hypocalcemia episodes were severe (Ca < 1.87 mmol/L), which is lower than that found in EVOLVE (18%) but comparable to other randomized trials (< 11%) [4, 9, 17]. The high rate of hypocalcemia in EVOLVE may be explained by the systematic assessment of laboratory values in a randomized clinical trial setting, which is more frequent than the recommended interval in routine clinical practice (i.e., 1–3 month interval). Also, the average baseline PTH level was > 700 pg/mL in EVOLVE vs. 600 pg/mL in this study.

Our findings differ from a North-American “real-world” analysis, where cinacalcet led to hypocalcemia in only 47% of patients and only 3% had severe hypocalcemia [18]. In comparing our largely Caucasian European cohort with the North American cohort of Brunelli et al. [18], PTH was similar (mean PTH of 650 pg/mL) but > 50% were African-American, and they had twice as many diabetic patients and the average age was about 10 years younger. All these factors may have substantially influenced treatment and management with cinacalcet. For example, it is thought that Afro-american dialysis patients exhibit less severe high-turnover bone disease at similar PTH levels as compared to Caucasians [19]. Thus, an average PTH level similar to that observed in our study would imply that there was less severe bone disease and a less pronounced “hungry-bone” like scenarios in the North American study. In addition, only 64% of the AROii cohort were prescribed concomitant vitamin D compared to 90% in the North American Cohort.

Prior observational and clinical studies reported that cinacalcet-induced hypocalcemia was transient, usually asymptomatic and self-limited [4, 9, 17, 18, 20, 21]. Indeed, hypocalcemia-induced serious adverse episodes were rare [4, 21] or absent [9, 17] in randomized clinical trials and there are few case reports describing symptomatic patients [22–24]. Our retrospective analysis is limited as hypocalcemia-related events were potentially not systematically captured. However, it is notable that we found no evidence for an increased number of cardiovascular, in particular arrhythmogenic events in the six months immediately following cinacalcet initiation, i.e. the period, when hypocalcemia event was likely to occur (median time to hypocalcemia event was 4 months).

Despite the transient and mostly asymptomatic nature of cinacalcet-induced hypocalcemia, it may still be important to prospectively identify those at risk. In this respect, our present analysis supports prior conclusions that patients with severe sHPT have higher baseline iPTH levels (a consistent predictor of hypocalcemia) and low baseline calcium [14, 18]. It is conceivable that low albumin also predicted hypocalcemia since total calcium rather than ionized calcium was analysed. However, in our sensitivity analyses, there were no differences in our findings when using corrected as opposed to uncorrected calcium. Cumulatively, these observations mirror studies on “hungry bone syndrome” after parathyroidectomy, where the severity of preoperative sHPT was also predictive of occurrence of hypocalcemia [10, 25]. In this study, we had two observations that were difficult to explain, namely having a catheter vascular access was associated with hypocalcemia and having low haemoglobin was associated with a lower risk of hypocalcemia. Although these may represent chance findings further studies need to take them into consideration when protocols are designed. Similarly, risk factors associated with severe hypocalcemia (catheter access, net ultrafiltration, CRP levels, and no paricalcitol use) were of uncertain clinical relevance as we had limited number of severe hypocalcaemia episodes to come to clear conclusions. Even though we assessed albumin-corrected levels, we cannot exclude that inflammation associated with catheter access and high CRP levels, affected protein-bound calcium levels. In addition, we cannot fully exclude that the hypocalcemia risk associated with catheter access might have resulted from inadvertent spillage of citrate locking solution into the systemic circulation. However, citrate locking of catheters was uncommon at the time of our baseline period (2007–2009).

Intrinsic and therapeutic responses to cinacalcet-induced hypocalcemia also closely mirrored data obtained in EVOLVE and in a North American analysis [11, 18] in that PTH levels failed to increase following the hypocalcemic event which is most likely due to persistent suppression of PTH release by the calcimimetic. Therapeutically, although a recent editorial cautioned against inertia [12], we once again observed that the most common response to hypocalcemia was to not change the therapeutic regimen. Indeed, in the about 40% of hypocalcemic patients who received a therapeutic intervention attempting to increase serum calcium, outcomes in terms of normalizing serum calcium were no different as compared to patients with no targeted intervention.

In addition to the limitations discussed above, the present analysis is constrained by a wide range of cinacalcet prescription. Indeed, we noted that, in particular in Eastern European countries, cinacalcet was mostly prescribed to patients with more severe sHPT (Online Resource 9). However, since 83% of our patients were from the Iberian Peninsula, it is unlikely that the different prescription behaviour in Eastern Europe has markedly skewed our analysis towards more severe sHPT cases. Second, our diagnosis of hypocalcemia was based on total rather than ionized serum calcium. While albumin-concentrations were available in 97% of the patients, albumin-corrected total calcium only correlates moderately with ionized serum calcium in advanced CKD [26] and others have concluded that uncorrected calcium is not inferior to corrected calcium in HD patients [15, 16]. Third, whereas short-term adjustments of dialysate calcium might not have been captured in severely hypocalcemic patients, data shown in Table 6 provides little evidence for any systematic trend towards higher dialysate calcium in those with hypocalcemia which is similar to our observations in EVOLVE [11].

In conclusion, in the present “real-world” analysis of incident European HD patients, initiation of cinacalcet led to hypocalcemia in two-thirds of the patients with 9% categorized as severe. Hypocalcemia was mostly asymptomatic, transient with and without targeted intervention to correct it and not associated with an increase in cardiovascular events or deaths. These observations lend further support to the current KDIGO guideline recommendation to avoid hypercalcemia in chronic dialysis patients but to tolerate mild and asymptomatic hypocalcemia in the context of calcimimetic treatment in order to avoid inappropriate calcium loading. However, vigilance will still be necessary in order not to miss the rare case of serious hypocalcemia with potential clinical significance.

## Electronic supplementary material

Below is the link to the electronic supplementary material.

Supplementary material 1 (PDF 421 kb)
