# Supplementary material for: Cinacalcet-induced hypocalcemia in a cohort of European haemodialysis patients: predictors, therapeutic approaches and outcomes
Source: J Nephrol. 2019 Dec 17;33(4):803–16. doi: 10.1007/s40620-019-00686-z (PMC7381480; doi:10.1007/s40620-019-00686-z)
Supplement: Supplementary file 1 — Supplementary material 1 (PDF 421 kb) [file 40620_2019_686_MOESM1_ESM.pdf]

Cinacalcet-induced hypocalcemia in a cohort of European haemodialysis patients: Predictors, therapeutic approaches and outcomes

Karly S. Louie, PhD[2], Clement Erhard, MSc[3], David C. Wheeler, MD[4], Peter Stenvinkel, MD PhD[5], Bruno Fouqueray, MD PhD[6], Jürgen Floege, MD[1]

[1]Division of Nephrology, RWTH University of Aachen, Germany

[2]Amgen Ltd., Uxbridge, United Kingdom

[3]Stanislas Limited, Uxbridge, United Kingdom

[4]Department of Nephrology, University College London, London, UK and George Institute for Global Health, Sydney, Australia.

[5]Division of Renal Medicine, Department of Clinical Science Technology and Intervention, Karolinska University Hospital, Karolinska Institutet, Stockholm, Sweden

[6]Amgen GmbH, Rotkreuz, Switzerland

Address for correspondence

Jürgen Floege MD

Division of Nephrology & Clinical Immunology

RWTH University of Aachen

Pauwelsstraße 30

D-52057 Aachen, Germany

Phone: +49-(0)241-8089530 / 531

E-mail: jfloege@ukaachen.de

Online Resource 1. Kaplan-Meier curve of time to first hypocalcemia episode, overall and by corrected serum Ca at time of cinacalcet initiation. Normal cCa levels were defined as low (2.1-<2.5 mmol/L), medium (2.5-<2.75 mmol/L) and high (≥2.75 mmol/L).

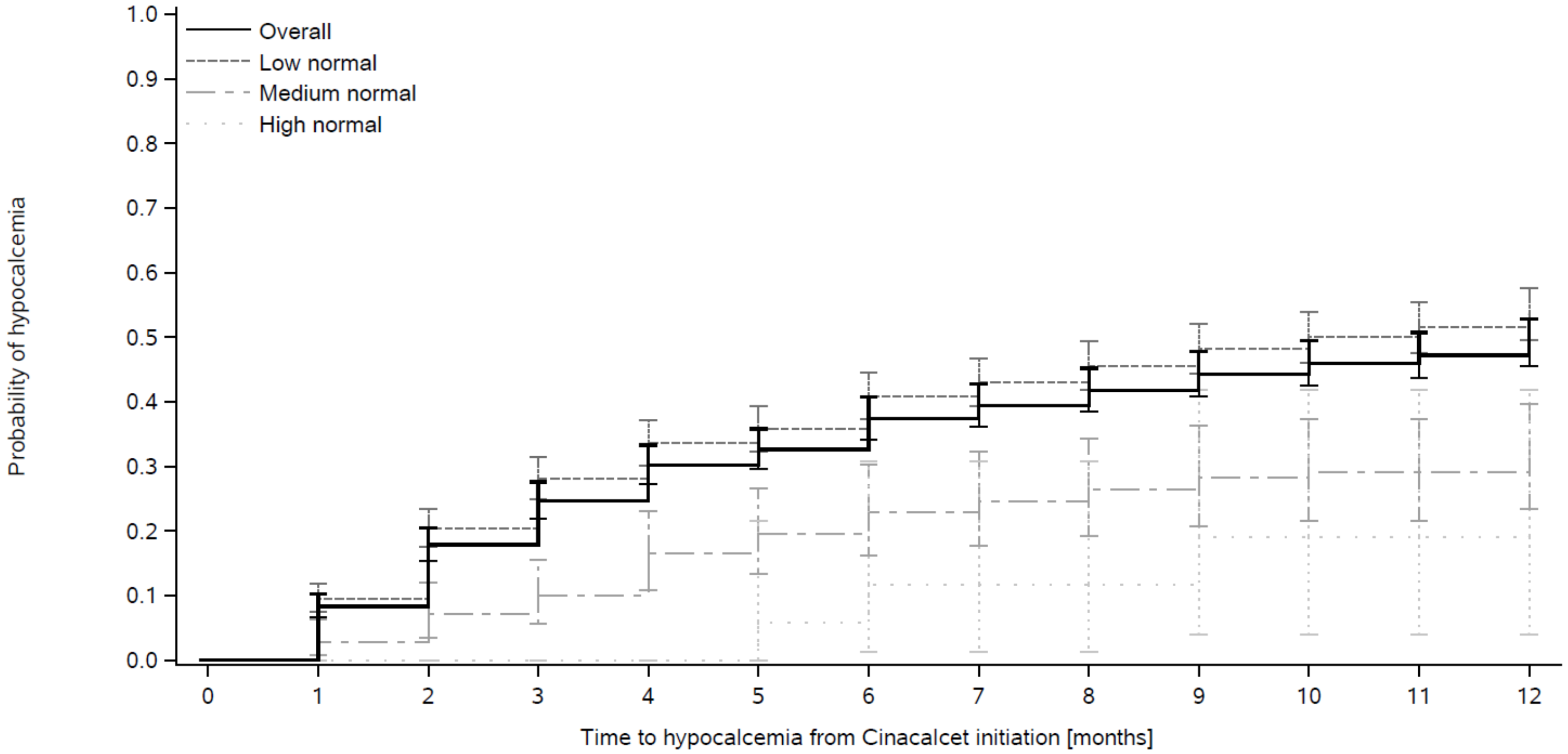

Subjects at risk

	0	1	2	3	4	5	6	7	8	9	10	11	12
Overall	878	878	805	721	659	568	507	445	407	368	336	310	290
Low normal	721	721	652	574	516	442	393	340	308	276	251	228	210
Medium normal	139	139	135	129	125	109	98	91	85	80	74	72	70
High normal	18	18	18	18	18	17	16	14	14	12	11	10	10

Online Resource 2. Kaplan-Meier curve of time to discontinuation in the 4 months following hypocalcaemia event, overall and by corrected Ca at time of hypocalcaemia episode and severity (mild Ca 2.0-<2.1 mmol/L, moderate Ca 1.87-<2.0 mmol/L, severe Ca<1.87 mmol/L)

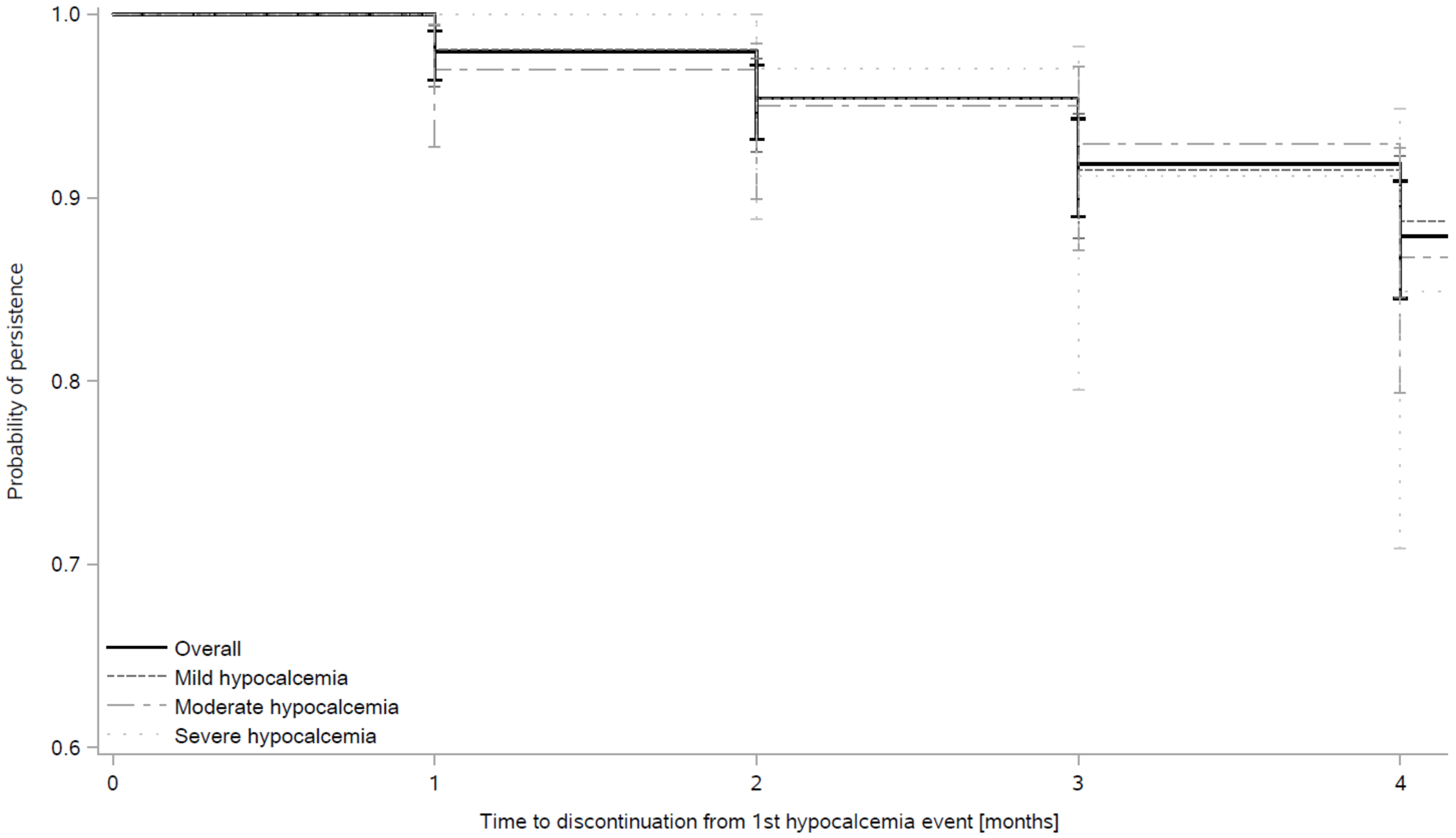

Online Resource 3. Baseline characteristics of patients who develop and do no develop hypocalcaemia 12 months after cinacalcet initiation according to corrected Ca

Baseline characteristics	No hypocalcemia N=483	Hypocalcaemia N=395	Patients who develop hypocalcemia within 12 months (N=395)		
			**Mild** Corrected Ca 2.00-<2.10 mmol/L N=261	**Moderate** Corrected Ca 1.87-<2.0 mmol/L N=100	**Severe** Corrected Ca <1.87 mmol/L N=34
**Patient age at index date [years][a]**	67 (55, 76)	66 (54, 76)	68 (53, 76)	66 (54, 75)	62 (54, 75)
**Male, No. (%)**	272 (56)	229 (58)	157 (60)	55 (55)	17 (50)
**Geographical area, No. (%)**					
Eastern Europe[b]	38 (8)	46 (12)	33 (13)	12 (12)	1 (3)
Western Europe[c]	47 (10)	25 (6)	17 (7)	5 (5)	3 (9)
Iberian peninsula[d]	398 (82)	324 (82)	211 (81)	83 (83)	30 (88)
**BMI [kg/m^2][a]**	27.0 (23.9, 30.7)	26.9 (24.5, 30.2)	26.9 (24.4, 30.2)	27.0 (24.5, 30.7)	27.8 (25.7, 30.5)
**BMI [kg/m^2] - category, No. (%)**					
<18.5	7 (1)	3 (1)	2 (1)	1 (1)	0 (0)
≥18.5 - <25	133 (28)	101 (26)	70 (27)	26 (26)	5 (15)
≥25 - <30	168 (35)	153 (39)	99 (38)	38 (38)	16 (47)
≥30	124 (26)	94 (24)	60 (23)	25 (25)	9 (27)
Missing	51 (11)	44 (11)	30 (12)	10 (10)	4 (12)
**Clinical history, No. (%)**					
Hospitalisation	229 (47)	224 (57)	152 (58)	51 (51)	21 (62)
Diabetes	152 (32)	130 (33)	89 (34)	29 (29)	12 (35)
Cancer	53 (11)	33 (8)	19 (7)	7 (7)	7 (21)
Cardiovascular disease	233 (48)	192 (49)	125 (48)	53 (53)	14 (41)
Fracture	22 (5)	22 (6)	13 (5)	7 (7)	2 (6)
**Dialysis duration per week [hours][a]**	12.0 (12.0, 12.4)	12.0 (12.0, 12.3)	12.0 (12.0, 12.3)	12.0 (12.0, 12.3)	12.0 (12.0, 12.0)
**Dialysis adequacy [Kt/V] - category, No. (%)**					
<1.2	52 (11)	64 (16)	38 (15)	19 (19)	7 (21)
≥1.2	388 (80)	301 (76)	202 (77)	73 (73)	26 (76)
No value recorded	43 (9)	30 (8)	21 (8)	8 (8)	1 (3)
**Dialysis vintage [years][a]**	1.9 (1.0, 3.1)	1.9 (1.0, 3.2)	1.9 (1.0, 3.1)	1.8 (0.9, 3.4)	1.8 (1.2, 2.7)
**Dialysate calcium [mmol/L][a]**	1.3 (1.3, 1.5)	1.3 (1.3, 1.5)	1.3 (1.3, 1.5)	1.3 (1.3, 1.5)	1.3 (1.3, 1.5)
**Catheter vascular access, No. (%)**					
Yes	90 (19)	107 (27)	63 (24)	30 (30)	14 (41)
No	378 (78)	274 (69)	188 (72)	67 (67)	19 (56)
Missing	15 (3)	14 (4)	10 (4)	3 (3)	1 (3)
**Net ultrafiltration [L][a]**	2.0 (1.4, 2.6)	2.2 (1.5, 2.7)	2.2 (1.5, 2.6)	2.2 (1.6, 2.8)	2.1 (1.4, 3.0)
**Blood haemoglobin [g/L][a]**	118 (110, 127)	120 (112, 128)	119 (112, 128)	121 (115, 130)	118 (113, 126)

**Serum albumin [g/L][a]**	40.0 (37.0, 42.5)	40.0 (37.0, 43.0)	40.0 (38.0, 43.0)	40.0 (36.0, 43.0)	39.0 (36.0, 42.6)
**Serum ferritin [µg/L][a]**	433 (263, 624)	391 (262, 596)	390 (264, 620)	389 (265, 582)	425 (244, 567)
**Serum CRP [mg/L][a]**	4.7 (1.7, 10.1)	4.9 (2.0, 10.0)	4.9 (2.0, 8.7)	5.3 (2.0, 12.0)	4.5 (2.3, 11.0)
**Serum calcium [mmol/L][a]**	2.4 (2.3, 2.5)	2.3 (2.2, 2.4)	2.3 (2.2, 2.4)	2.2 (2.2, 2.3)	2.2 (2.2, 2.3)
**Serum calcium [mmol/L] - category, No. (%)**					
<2.10	16 (3)	27 (7)	17 (7)	6 (6)	4 (12)
≥2.10 - <2.50	368 (76)	336 (85)	221 (85)	87 (87)	28 (82)
≥2.50 - <2.75	87 (18)	29 (7)	20 (8)	7 (7)	2 (6)
≥2.75	12 (3)	3 (1)	3 (1)	0 (0)	0 (0)
Missing	0 (0)	0 (0)	0 (0)	0 (0)	0 (0)
**Serum phosphate [mmol/L][a]**	1.7 (1.4, 2.0)	1.7 (1.4, 2.0)	1.7 (1.4, 2.0)	1.7 (1.4, 2.0)	1.8 (1.5, 2.0)
**Serum PTH [ng/L][a]**	551 (411, 783)	619 (462, 841)	605 (455, 825)	673 (462, 914)	628 (522, 1072)
**Calcium, albumin-corrected [mmol/L][a]**	2.4 (2.3, 2.5)	2.3 (2.2, 2.4)	2.3 (2.2, 2.4)	2.3 (2.2, 2.4)	2.3 (2.2, 2.4)
**Calcium, albumin-corrected [mmol/L], No. (%)**					
<2.10	0 (0)	0 (0)	0 (0)	0 (0)	0 (0)
≥2.10 - <2.50	369 (76)	352 (89)	227 (87)	93 (93)	32 (94)
≥2.50 - <2.75	99 (21)	40 (10)	31 (12)	7 (7)	2 (6)
≥2.75	15 (3)	3 (1)	3 (1)	0 (0)	0 (0)
**Drug use, No. (%)**					
Calcitriol/alfacalcidol	127 (26)	101 (26)	63 (24)	29 (29)	9 (27)
Paricalcitol	192 (40)	183 (46)	125 (48)	42 (42)	16 (47)
**Phosphate binder use, No. (%)**					
Non-calcium based only	223 (46)	155 (39)	106 (41)	36 (36)	13 (38)
Calcium based only	46 (10)	43 (11)	26 (10)	15 (15)	2 (6)
Both calcium based and non-calcium based	113 (23)	88 (22)	62 (24)	18 (18)	8 (24)
None	101 (21)	109 (28)	67 (26)	31 (31)	11 (32)

[a] Median (Q1, Q3)

[a] Eastern Europe: Czech Republic, Hungary, Poland, Romania, Russia, Serbia, Slovak Republic, Slovenia and Turkey

[b] Western Europe: France, Ireland, Italy, and the United Kingdom

[c] Iberian Peninsula: Portugal and Spain

Online Resource 4. Baseline predictors of time to first hypocalcaemia episode (corrected Ca) in the first 12 months after cinacalcet initiation

	**Hypocalcaemia vs. no hypocalcaemia** Corrected Ca <2.10 mmol/L vs. Corrected Ca ≥2.10 mmol/L		
**Baseline Predictor**	No. of hypocalcaemia events/ Total no. of subjects (%)	Hazard ratio (95 % CI)	p-value
**Geographical area**			0.003
Iberian peninsula[a]	324/722 (44.9)	Reference	
Eastern Europe[b]	46/84 (54.8)	1.73 (1.16, 2.57)	
Western Europe[c]	25/72 (34.7)	0.70 (0.46, 1.06)	
**Hospitalisation**			0.018
No	171/425 (40.2)	Reference	
Yes	224/453 (49.4)	1.28 (1.04, 1.58)	
**Smoking status**			0.008
Non smoker	124/310 (40.0)	Reference	
Current	41/97 (42.3)	1.05 (0.73, 1.50)	
Former	62/126 (49.2)	1.47 (1.08, 2.00)	
Missing information	168/345 (48.7)	1.44 (1.13, 1.83)	
**Catheter vascular access**			0.002
No	274/652 (42.0)	Reference	
Yes	107/197 (54.3)	1.54 (1.21, 1.95)	
Missing	14/29 (48.3)	1.19 (0.63, 2.25)	
**Net Ultrafiltration [L]**			0.026
Q1	92/227 (40.5)	Reference	
Q2	88/215 (40.9)	1.03 (0.76, 1.38)	
Q3	118/233 (50.6)	1.37 (1.04, 1.81)	
Q4	97/203 (47.8)	1.40 (1.04, 1.87)	
**Haemoglobin [g/dL]**			0.013
≥12	201/425 (47.3)	Reference	
≥10 - <12	181/408 (44.4)	0.87 (0.71, 1.06)	
<10	13/45 (28.9)	0.44 (0.25, 0.78)	
**PTH [ng/L]**			0.043
<600	188/458 (41.0)	Reference	
600 - <1000	141/279 (50.5)	1.33 (1.06, 1.65)	
≥1000	66/141 (46.8)	1.15 (0.85, 1.55)	
**Paricalcitol use**			0.038
Yes	183/375 (48.8)	Reference	
No	212/503 (42.1)	0.81 (0.66, 0.99)	

PTH, parathyroid hormone

[a] Eastern Europe: Czech Republic, Hungary, Poland, Romania, Russia, Serbia, Slovak Republic, Slovenia and Turkey

[b] Western Europe: France, Ireland, Italy, and the United Kingdom

[c] Iberian Peninsula: Portugal and Spain

Online Resource 5. Baseline predictors of time to first severe hypocalcaemia event according to corrected Ca in the first 12 months after cinacalcet initiation

Predictor	Severe hypocalcaemia vs. No hypocalcaemia<br>Corrected Ca <1.87 mmol/L vs. Corrected Ca ≥2.10 mmol/L		
	No. of severe hypocalcaemia events/ Total no. of subjects (%)	Hazard ratio (95 % CI)	p-value
**Catheter vascular access**			0.02
No	19/397 (4.8)	Reference	
Yes	14/104 (13.5)	2.77 (1.36, 5.65)	
No data	1/16 (6.3)	1.52 (0.20, 11.37)	

Online Resource 6. Treatment characteristics of chronic haemodialysis patients at time of cinacalcet initiation according to corrected Ca levels

		Hypocalcaemia within 12 months after cinacalcet initiation<br>Corrected Ca at time of cinacalcet initiation		
**At cinacalcet initiation**	**No hypocalcaemia within 12 months after cinacalcet initiation**<br>N=483	**Overall**<br>N=395	**Low normal Corrected Ca**<br>2.10-<2.5 mmol/L<br>N=352	**Moderate to high normal corrected Ca**<br>≥2.5 mmol/L<br>N=43
**Cinacalcet**				
Average daily dose [mg/day], median (Q1, Q3)	30.0 (12.9, 30.0)	30.0 (30.0, 30.0)	30.0 (28.3, 30.0)	30.0 (30.0, 30.0)
**Calcitriol/Alfacalcidol**				
Use, n (%)	107 (22)	88 (22)	80 (23)	8 (19)
Average daily dose [µg/day], median (Q1, Q3)	0.4 (0.2, 0.5)	0.3 (0.2, 0.4)	0.3 (0.2, 0.4)	0.3 (0.1, 0.8)
New users, n (%)	4 (4)	2 (2)	2 (3)	0 (0)
Continuing users, n (%)	103 (96)	86 (98)	78 (98)	8 (100)
Stable dose, n (%)	85 (83)	71 (83)	64 (82)	7 (88)
Up-titration, n (%)	12 (12)	6 (7)	6 (8)	0 (0)
Down-titration, n (%)	6 (6)	9 (10)	8 (10)	1 (13)
**Paricalcitol**				
Use, n (%)	169 (35)	162 (41)	144 (41)	18 (42)
Average daily dose [µg/day], median (Q1, Q3)	0.9 (0.6, 1.3)	0.9 (0.6, 1.4)	0.9 (0.6, 1.4)	0.8 (0.6, 1.1)
New users, n (%)	5 (3)	14 (9)	14 (10)	0 (0)
Continuing users, n (%)	164 (97)	148 (91)	130 (90)	18 (100)
Stable dose, n (%)	115 (70)	113 (76)	101 (78)	12 (67)
Up-titration, n (%)	31 (19)	19 (13)	16 (12)	3 (17)
Down-titration, n (%)	18 (11)	16 (11)	13 (10)	3 (17)
**Calcium-based phosphate binder**				
Use, n (%)	102 (21)	95 (24)	89 (25)	6 (14)
Average daily dose [mg/day], median (Q1, Q3)	2000 (1016, 3960)	1980 (1143, 3000)	1980 (1143, 3000)	2133 (500, 4200)
New users, n (%)	3 (3)	3 (3)	3 (3)	0 (0)
Continuing users, n (%)	99 (97)	92 (97)	86 (97)	6 (100)
Stable dose, n (%)	87 (88)	82 (89)	77 (90)	5 (83)
Up-titration, n (%)	7 (7)	5 (5)	5 (6)	0 (0)
Down-titration, n (%)	5 (5)	5 (5)	4 (5)	1 (17)
**Dialysate calcium [mmol/L]**				
Missing	13 (3)	18 (5)	14 (4)	4 (9)
≤1.00	2 (0)	2 (1)	2 (1)	0 (0)
>1.00 to <1.25	0 (0)	0 (0)	0 (0)	0 (0)
≥1.25 to <1.50	248 (51)	204 (52)	183 (52)	21 (49)
≥1.50	220 (46)	171 (43)	153 (43)	18 (42)

Stable dose, n (%)	435 (93)	355 (94)	320 (95)	35 (90)
Up-titration, n (%)	12 (3)	8 (2)	8 (2)	0 (0)
Down-titration, n (%)	21 (4)	13 (3)	9 (3)	4 (10)

Online Resource 7. Management of hypocalcaemia within 90 days following first hypocalcaemia episode according to severity

	Overall			Albumin corrected Calcium at time of first hypocalcaemia								
				**Mild** Corrected Ca 2.0- <2.10 mmol/L			**Moderate** Corrected Ca 1.87-<2.00 mmol/L			**Severe** Corrected Ca <1.87 mmol/L		
**Management of hypocalcaemia following first hypocalcaemia event**	No. at risk	n	%	No. at risk	n	%	No. at risk	n	%	No. at risk	n	%
**Dialysate calcium**												
Increased dialysate calcium	395	63	16	261	37	14	134	26	19	395	63	16
Stable dialysate calcium	395	304	77	261	204	78	134	100	75	395	304	77
Decreased dialysate calcium	395	7	2	261	5	2	134	2	2	395	7	2
**Cinacalcet**												
Increased cinacalcet dose	395	49	12	261	39	15	134	10	8	395	49	12
Stable cinacalcet dose	395	237	60	261	157	60	134	80	60	395	237	60
Discontinued	395	32	8	261	22	8	134	10	8	395	32	8
Reduced cinacalcet dose	395	76	19	261	43	17	134	33	25	395	76	19
**Calcitriol/Alfacalcidol**												
Initiated	307	25	8	202	11	5	105	14	13	307	25	8
Up-titrated	88	22	25	59	14	24	29	8	28	88	22	25
Initiated or up-titrated	395	47	12	261	25	10	134	22	16	395	47	12
Stable	88	53	60	59	37	63	29	16	55	88	53	60
Down-titrated	88	9	10	59	6	10	29	3	10	88	9	10
Discontinued	88	4	5	59	2	3	29	2	7	88	4	5
**Paricalcitol**												
Initiated	250	45	18	162	26	16	88	19	22	250	45	18
Up-titrated	145	44	30	99	32	32	46	12	26	145	44	30
Initiated or up-titrated	395	89	23	261	58	22	134	31	23	395	89	23
Stable	145	71	49	99	47	48	46	24	52	145	71	49
Down-titrated	145	22	15	99	14	14	46	8	17	145	22	15
Discontinued	145	8	6	99	6	6	46	2	4	145	8	6
**Calcium-based phosphate binder**												
Initiated	288	38	13	194	24	12	94	14	15	288	38	13
Up-titrated	107	15	14	67	10	15	40	5	13	107	15	14
Initiated or up-titrated	395	53	13	261	34	13	134	19	14	395	53	13
Stable	107	82	77	67	51	76	40	31	78			
Down-titrated	107	8	8	67	5	8	40	3	8			
Discontinued	107	2	2	67	1	2	40	1	3			
**Any expected responses[1]**												
No treatment intervention	395	145	37	261	109	42	134	36	27			
Any treatment intervention	395	250	63	261	152	58	134	98	73			

**Any responses[2]**									
No treatment intervention	395	102	26	261	76	29	134	26	19
Any treatment intervention	395	293	74	261	185	71	134	108	81

[1]Any expected treatment response = for Cinacalcet, discontinued or reduced; for Dialysate Calcium: increased calcium; for other drugs: initiated or up-titrated

[2]Any treatment = any of the changes listed in table

Online Resource 8. Characteristics of patients who discontinued within 4 months following hypocalcemia event and those who don't

Characteristics	All patients (N=610)	Overall no. of patients who did not discontinue within 4 months following hypocalcemia event (N=544)	Overall no. of patients who discontinued within 4 months following hypocalcemia event (N=66)
**Patient age at index date [years][a]**	67 (54, 76)	66 (54, 76)	72 (63, 75)
**Gender, No. (%)**			
Male	352 (58)	313 (58)	39 (59)
Female	257 (42)	230 (42)	27 (41)
Missing	1 (0)	1 (0)	0 (0)
**Geographical area, No. (%)**			
Eastern Europe	56 (9)	48 (9)	8 (12)
Western Europe	41 (7)	36 (7)	5 (8)
Iberian peninsula	513 (84)	460 (85)	53 (80)
**Period pre-KDIGO, No. (%)**			
Yes	134 (22)	118 (22)	16 (24)
No	476 (78)	426 (78)	50 (76)
Missing	0 (0)	0 (0)	0 (0)
**BMI [kg/m^2] at baseline[a]**	27.0 (24.3, 30.2)	27.0 (24.4, 30.3)	26.7 (23.5, 29.4)
**BMI [kg/m^2] at baseline - category, No. (%)**			
<18.5	7 (1)	7 (1)	0 (0)
≥18.5 - <25	162 (27)	138 (25)	24 (36)
≥25 - <30	232 (38)	209 (38)	23 (35)
≥30	148 (24)	134 (25)	14 (21)
Missing	61 (10)	56 (10)	5 (8)
**Clinical history at baseline, No. (%)**			
Hospitalisation	310 (51)	280 (51)	30 (45)
Diabetes	194 (32)	169 (31)	25 (38)
Cancer	59 (10)	50 (9)	9 (14)
Cardiovascular disease	304 (50)	272 (50)	32 (48)
Fracture	29 (5)	23 (4)	6 (9)
**Dialysis frequency [times/wk] at baseline - category, No. (%)**			
3	601 (99)	536 (99)	65 (98)
4	5 (1)	4 (1)	1 (2)
Missing	0 (0)	0 (0)	0 (0)
Other	4 (1)	4 (1)	0 (0)

**Dialysis duration per week [hours] at baseline[a]**	12.0 (12.0, 12.3)	12.0 (12.0, 12.3)	12.0 (12.0, 12.4)
**Dialysis adequacy [Kt/V] at baseline - category, No. (%)**			
<1.2	88 (14)	82 (15)	6 (9)
≥1.2	474 (78)	420 (77)	54 (82)
No value recorded	48 (8)	42 (8)	6 (9)
**Dialysis vintage [years][a]**	1.8 (1.0, 3.0)	1.8 (1.0, 3.0)	1.9 (1.2, 3.0)
**Dialysis vintage [years] - category, No. (%)**			
<1	158 (26)	144 (26)	14 (21)
≥1 - ≤5	427 (70)	376 (69)	51 (77)
>5	25 (4)	24 (4)	1 (2)
Missing	0 (0)	0 (0)	0 (0)
**Dialysate calcium [mmol/L] at baseline[a]**	1.3 (1.3, 1.5)	1.3 (1.3, 1.5)	1.5 (1.3, 1.5)
**Dialysate calcium [mmol/L] at baseline - category, No. (%)**			
<1.5	305 (50)	278 (51)	27 (41)
≥1.5	288 (47)	254 (47)	34 (52)
No value recorded	17 (3)	12 (2)	5 (8)
**Cathetar vascular access at baseline, No. (%)**			
Yes	158 (26)	139 (26)	19 (29)
No	434 (71)	392 (72)	42 (64)
Missing	18 (3)	13 (2)	5 (8)
**Net ultrafiltration [L] at baseline[a]**	2.1 (1.4, 2.7)	2.1 (1.5, 2.7)	1.9 (1.4, 2.6)
**Blood hemoglobin [g/L] at hypocalcemia[a]**	116 (108, 124)	116 (108, 124)	117 (110, 126)
**Serum albumin [g/L] at hypocalcemia[a]**	39.0 (36.9, 41.0)	39.0 (37.0, 41.1)	38.0 (35.0, 40.2)
**Serum ferritin [µg/L] at hypocalcemia[a]**	400 (265, 587)	397 (265, 580)	466 (277, 624)
**Serum CRP [mg/L] at hypocalcemia[a]**	5.7 (1.5, 12.0)	5.0 (1.3, 11.4)	8.1 (4.5, 20.3)
**Serum calcium [mmol/L] at hypocalcemia[a]**	2.0 (2.0, 2.1)	2.0 (2.0, 2.1)	2.0 (2.0, 2.1)
**Serum calcium [mmol/L] at hypocalcemia - category, No. (%)**			
<1.87	54 (9)	47 (9)	7 (11)
≥1.87 - <2.00	141 (23)	126 (23)	15 (23)
≥2.00 - <2.10	415 (68)	371 (68)	44 (67)
≥2.10 - <2.50	0 (0)	0 (0)	0 (0)
≥2.50 - <2.75	0 (0)	0 (0)	0 (0)
≥2.75	0 (0)	0 (0)	0 (0)
Missing	0 (0)	0 (0)	0 (0)

**Serum phosphate [mmol/L] at hypocalcemia**[a]	1.5 (1.2, 1.7)	1.5 (1.2, 1.7)	1.4 (1.1, 1.6)
**Serum phosphate [mg/dL] at hypocalcemia - category, No. (%)**			
<1.13	104 (17)	89 (16)	15 (23)
≥1.13 - ≤1.78	383 (63)	343 (63)	40 (61)
>1.78	122 (20)	111 (20)	11 (17)
Missing	1 (0)	1 (0)	0 (0)
**Serum PTH [ng/L] at hypocalcemia**[a]	380 (236, 600)	393 (241, 611)	291 (172, 469)
**Calcium, albumin-corrected [mmol/L] at hypocalcemia**[a]	2.1 (2.0, 2.1)	2.1 (2.0, 2.1)	2.1 (2.0, 2.1)
**Calcium, albumin-corrected [mmol/L] at hypocalcemia, No. (%)**			
<1.87	21 (3)	20 (4)	1 (2)
≥1.87 - <2.00	62 (10)	54 (10)	8 (12)
≥2.00 - <2.10	206 (34)	188 (35)	18 (27)
≥2.10 - <2.50	102 (17)	85 (16)	17 (26)
≥2.50 - <2.75	0 (0)	0 (0)	0 (0)
≥2.75	0 (0)	0 (0)	0 (0)
Missing	219 (36)	197 (36)	22 (33)
**Drug use at hypocalcemia, No. (%)**			
Calcitriol/alfacalcidol	135 (22)	121 (22)	14 (21)
Paricalcitol	235 (39)	216 (40)	19 (29)
Cardiovascular medication	514 (84)	459 (84)	55 (83)
**Phosphate binder use at hypocalcemia, No. (%)**			
Non-calcium based only	272 (45)	242 (44)	30 (45)
Calcium based only	68 (11)	62 (11)	6 (9)
Both calcium based and non-calcium based	135 (22)	123 (23)	12 (18)
None	135 (22)	117 (22)	18 (27)

[a] Median (Q1, Q3)

Online Resource 9. Baseline PTH by country at time of cinacalcet initiation

Country	Total no. of patients	PTH<600 pg/L n (%)		PTH 600-<1000 pg/mL n (%)		PTH ≥1000 pg/mL n (%)	
Czech Republic	39	24	61.5	10	25.6	5	12.8
France	7	0	0.0	4	57.1	3	42.9
Hungary	10	1	10.0	3	30.0	6	60.0
Ireland	1	0	0.0	0	0.0	1	100.0
Italy	35	22	62.9	6	17.1	7	20.0
Portugal	251	72	28.7	125	49.8	54	21.5
Romania	1	1	100.0	0	0.0	0	0.0
Russia	7	0	0.0	3	42.9	4	57.1
Serbia	1	1	100.0	0	0.0	0	0.0
Slovenia	6	1	16.7	2	33.3	3	50.0
Spain	499	343	68.7	120	24.0	36	7.2
Turkey	17	2	11.8	0	0.0	15	88.2
United Kingdom	31	9	29.0	10	32.3	12	38.7
All countries	905	476	52.6	283	31.3	146	16.1

PTH, parathyroid hormone
